# Neural Remodeling Across Cancer Evolution: Temporal, Spatial, Cellular, and Functional Principles of Tumor–Nerve Ecosystems

**DOI:** 10.3390/cancers18213504

**Published:** 2026-10-30

**Authors:** Noura A. A. Ebrahim, Rasha Aziz Attia Salama, Soliman M. A. Soliman

**Affiliations:** 1 Oncologic Pathology Department, National Cancer Institute, Cairo University, Giza P.O. Box 12613, Egypt; 2 Department of Community Medicine, College of Medicine, Ras Al Khaimah Medical and Health Sciences University, Ras Al Khaimah 11172, United Arab Emirates; 3 Chemistry Department, Faculty of Science, Cairo University, Giza P.O. Box 12613, Egypt; sabdellatif@sci.cu.edu.eg

**Keywords:** cancer neuroscience, tumor–nerve interactions, neural remodeling, neural microdomain, neuroimmune crosstalk, spatial transcriptomics, perineural invasion, clinical translation

## Abstract

Cancer neuroscience now asks not only whether tumors contain nerves, but how neural architecture, neuronal and glial states, and neural output change through cancer evolution. This critical narrative review integrates evidence from precursor lesions, primary tumors, invasion, metastasis, and treatment-associated states, while distinguishing neural remodeling from axonogenesis, neurogenesis, perineural invasion, and direct synaptic integration. We compare trophic, axon-guidance, metabolic, extracellular-vesicle, stromal, and neuroimmune mechanisms and emphasize that the direction of neural effects depends on stage, neuronal subtype, receptor context, and tissue immunity. The review operationalizes the proposed neural microdomain as a spatially bounded, measurable unit linking neural identity and topology to neighboring tumor, immune, stromal, and vascular states. It also separates neural abundance, neural state, and neural activity, and applies a consistent evidence taxonomy spanning anatomical, molecular, preclinical functional, human observational, and interventional evidence. Recent reports involving treatment-associated remodeling, systemic circuits, metastatic adaptation, and neural biomarkers are treated as emerging or preclinical where appropriate. We provide practical criteria for microdomain measurement, patient-level quantitative analysis, controls for denervation-related confounding, and distinctions among prognostic, predictive, and response biomarkers. The central translational conclusion is that neural interventions should be stage- and circuit-specific, biomarker-supported, and designed to preserve essential physiological neural functions.

## Simple Summary

Many cancers contain nerves, but the presence of nerves alone does not reveal whether they help or restrain the disease. Research shows that tumor cells, immune cells, support cells, and nerves can change one another over time. This review explains how these interactions differ between early lesions, established tumors, metastases, and treated cancers. It proposes a practical way to map small tumor regions where neural fibers meet defined tumor and neighboring cell states, while distinguishing nerve amount, molecular state, and activity. It also separates observations in patient samples from mechanisms tested in laboratory models and clinical trials. This approach may help researchers compare studies consistently and identify neural targets that are measurable in patients and safe enough to investigate without disrupting essential nerve functions.

## 1. Introduction

The tumor microenvironment (TME) is an evolving ecosystem in which malignant cells interact with fibroblasts, endothelial and lymphatic compartments, the extracellular matrix (ECM), metabolites, immune populations, and neural structures. The nervous system is unusual within this ecosystem because neural signaling combines spatial precision, rapid temporal dynamics, electrical excitability, local neurotransmitter release, and systemic control of organ physiology. Cancer neuroscience has therefore shifted from the descriptive question of whether nerves are present in tumors toward a more demanding question: how do tumors recruit, redistribute, reprogram, or co-opt neural circuits as malignancy emerges, progresses, disseminates, and is treated [1,2,3,4,5,6,7,8]?

Two complementary perspectives frame the field: cancer may exploit or become dependent on neural circuitry, and neural components intersect with multiple cancer hallmarks and immune programs [9,10]. Recent reviews have surveyed therapeutic opportunities, neural–tumor microenvironment reprogramming, and repurposing of neuroactive drugs [11,12,13,14]. These syntheses provide essential conceptual maps, while primary studies demonstrate context-specific effects across prostate, gastric, breast, pancreatic, colorectal, lung, liver, skin, and central nervous system malignancies [1,2,15,16,17,18,19,20,21,22,23,24]. Together, this literature establishes that tumor–neural communication is neither confined to one organ nor reducible to a single neurotransmitter pathway; the relevant target may be a nerve projection, a reprogrammed neuronal state, glia, an immune intermediary, or a local circuit.

The major challenge is interpretive. A higher density of nerve fibers does not establish that nerves drive tumor progression; a receptor–ligand pair detected in a transcriptomic dataset does not establish signal delivery; and denervation does not necessarily isolate a neural mechanism because nerve interruption can change injury responses, vascular tone, inflammation, and tissue repair. Conversely, apparently negative or paradoxical findings may reflect differences in disease stage, neural branch, receptor repertoire, host tissue, or experimental mode [3,4,5,6,7,8].

The present review makes five linked dimensions explicit: temporal emergence, neuronal and glial identity, spatial architecture, functional consequence, and causal perturbation. Its contribution is not the general claim that tumors interact with nerves, nor a new label for neural niches. It is an operational and evidence-calibrated workflow: define what neural feature changed, locate it in a specific disease stage and spatial neighborhood, determine whether abundance, state, or activity was measured, and state what level of causality the design can support. The neural microdomain is specified through reportable spatial and cellular criteria, and observations from human tumors are kept distinct from mechanisms established by perturbation in experimental models (Table 1).

The overlap with recent reviews is substantial, and the present review does not claim novelty from discussing neural addiction, neural hijacking, neuroimmune crosstalk, or therapeutic neuromodulation alone [5,6,7,8,9,10,11,12,13,14]. The “neural addiction” and cancer-hallmarks perspectives describe broad conceptual relationships; the therapeutic reviews emphasize repurposing and intervention; and recent neural–tumor microenvironment syntheses discuss reprogramming, systemic circuits, and perineural invasion. These approaches complement the present review rather than replace it. Our distinctive contribution is to turn the broader concepts into a practical comparison framework that links (i) time and disease stage, (ii) projection and glial identity, (iii) spatially measured neural microdomains, (iv) separate readouts of abundance, state, and activity, and (v) an explicit evidence label for each claim. The framework is designed to expose rather than erase contradictory findings and to clarify what would be needed to move a candidate mechanism toward a reproducible patient biomarker or an intervention trial.

## 2. Scope and Evidence Framework

This critical narrative review used targeted searches of PubMed/MEDLINE and publisher-indexed records, supplemented by backward and forward citation tracking. Search terms covered cancer neuroscience, tumor–nerve interactions, neural remodeling and innervation, axonogenesis, perineural invasion, neuroimmune crosstalk, neural microdomains, denervation, Trace-n-Seq, spatial transcriptomics, treatment-associated remodeling, and neural biomarkers, combined with terms for pancreatic, colorectal, breast, melanoma, lung, liver, prostate, gastric, and brain cancers. We prioritized mechanistic primary studies, human tissue and cohort studies, methodological reports, and recent reviews relevant to chronology, neural identity, spatial organization, causal evidence, or clinical readiness, considering the literature through 31 August 2026. This was not a systematic review; no protocol was registered, independent duplicate screening and formal risk-of-bias assessment were not performed, and no meta-analysis was undertaken.

For consistency, five evidence domains are distinguished. Anatomical evidence establishes where neural structures are located. Molecular evidence identifies altered ligands, receptors, neuronal states, or associated programs. Preclinical functional evidence demonstrates that perturbing a neural component changes a phenotype in a controlled model. Human observational evidence establishes relevance in patient material or clinical datasets but does not by itself establish causality. Prospective or interventional clinical evidence is the highest translational level and is currently sparse. These categories should be interpreted as cumulative rather than mutually exclusive.

The same principle applies to technologies. Histology can establish morphology but usually cannot establish function. Single-cell RNA sequencing can resolve cellular states but can miss distal neuronal somata and cannot directly measure neurotransmitter release. Spatial transcriptomics provides molecular geography, but its spatial resolution and sensitivity vary by platform. Retrograde tracing establishes projection identity, not functional dependence. Electrophysiology, calcium imaging, optogenetics, chemogenetics, genetic perturbation, receptor blockade, and rescue experiments can provide stronger causal evidence, but each has model-specific limitations [17,18,25,26,27,28,29,30,31,32,33].

## 3. Conceptual Boundaries: What Should Be Called Neural Remodeling?

Terminological precision is essential because the field contains several partially overlapping phenomena. In this review, neural remodeling refers to disease-associated changes in neural architecture, neuronal identity, neural connectivity, neural activity, or glial state. Axonogenesis refers specifically to growth, sprouting, branching, or extension of axons. Tumor innervation refers to neural projections entering or surrounding a tumor, without implying their origin or function. Neurogenesis should be reserved for evidence of neuronal generation or expansion and should not be used simply to describe axonal sprouting. Perineural invasion (PNI) refers to malignant cell invasion involving the nerve sheath or nerve microenvironment according to pathological criteria; it is a route of tumor spread rather than a synonym for neural remodeling [4,34,35].

A second important boundary concerns the CNS. Gliomas and some brain metastases arise within tissue in which neuronal activity is itself a major physiological variable. Direct synaptic signaling, membrane depolarization, and electrical coupling are therefore mechanistically distinct from peripheral axonogenesis. Glioma cells can receive glutamatergic synaptic input and integrate into functional neural circuits; human-induced-pluripotent-stem-cell-derived co-cultures have also demonstrated cholinergic neuron-to-glioblastoma synaptic interactions [17,18,26].

A third boundary concerns neural guidance versus PNI. Tumor cells may use axonal structures as directional substrates or respond to axon-guidance receptors without satisfying histopathological criteria for PNI. Conversely, PNI can involve Schwann-cell-mediated cancer-cell migration and tissue injury programs that are not equivalent to physiologic synaptic signaling. Schwann cells are particularly important because they can provide a permissive interface between nerves and invasive cancer cells, and their behavior may change before overt malignant invasion is established [34,35].

These distinctions are not semantic refinements alone. They determine the appropriate endpoint. A study of axonogenesis should quantify branching and projection geometry; a study of PNI should define pathological invasion; a study of neuronal reprogramming requires projection-aware neuronal profiling; and a study of synaptic integration requires functional measurement. Combining these endpoints without operational definitions can produce apparent contradictions that are methodological rather than biological.

## 4. Temporal Emergence: Neural Remodeling from Premalignancy to Recurrence

### 4.1. Premalignant and Metaplastic States

Some of the clearest experimental evidence that neural remodeling can precede overt malignancy comes from pancreatic carcinogenesis. Neuroplastic changes were detected during precursor stages, demonstrating that the neural compartment can respond to tissue transformation before invasive PDAC is established. Early pancreatic lesions may therefore act as active organizers of their neural environment rather than simply as passive recipients of nerves [36,37].

Recent multimodal work has made this concept mechanistically more precise. A 2026 study implicated ganglioside-mediated neural-acinar crosstalk as a molecular interface between epithelial lipid remodeling and neuritogenesis during ADM and PanIN development [38]. Independently, Netrin-1 expressed by metaplastic and precursor pancreatic lesions promoted sympathetic axon growth and branching through neuronal DCC; inhibition of Netrin-1 reduced local remodeling but accelerated PanIN formation in the experimental model, supporting a stage-specific protective role for lesion-associated sympathetic plasticity during this early phase [37].

The same ligand can therefore participate in biologically different circuits at different times. In established PDAC, Netrin-1 and its receptor NEO1 can promote tumor-cell growth, epithelial–mesenchymal transition (EMT), stem-like programs, and sympathetic innervation, with Ntn1 deletion reducing these features in experimental models. These apparently contrasting findings can be reconciled provisionally by considering ligand source, receptor, disease stage, cellular target, and neural output as components of a context-dependent circuit rather than as isolated pathway properties [39].

This stage dependence cautions against assuming that “more innervation” is intrinsically oncogenic. The recent pancreatic literature illustrates at least three possibilities: neural remodeling can restrain precursor progression; the same broader neural compartment can become tumor-supportive after malignant transition; or the neural phenotype can be a parallel adaptation with limited causal influence. Disease interception strategies must therefore avoid treating precancerous and established tumors as biologically interchangeable.

### 4.2. Established Primary Tumors

Once malignancy is established, neural remodeling can involve increased or redistributed fibers, altered branch composition, transcriptional reprogramming of tumor-innervating neurons, changes in neurotransmitter output, and reciprocal remodeling of tumor and stromal states. The Trace-n-Seq approach demonstrated that neurons innervating PDAC can be molecularly distinct from neurons innervating healthy pancreas, illustrating why bulk tumor sequencing provides an incomplete representation of the neural compartment [25].

The temporal interpretation of established disease should also account for neural degeneration and apparently divergent colorectal findings. One 2025 study reported that greater nerve density was associated with poorer outcome and that denervation suppressed tumor growth, whereas another reported local sympathetic neurodegeneration and found that sympathetic ablation exacerbated progression in an experimental model, with evidence that sympathetic signaling supported chemokine-mediated CD8+ T-cell recruitment. The direction of effect therefore cannot be generalized across colorectal cohorts and models; differences in disease stage, neural compartment, host immunity, and experimental perturbation may contribute [20,21].

### 4.3. Dissemination, Treatment, and Recurrence

Metastatic disease introduces a second temporal problem: the neuronal environment of the target organ differs from that of the primary tumor. Neural biology may therefore influence metastatic organotropism before arrival, provide a niche after dissemination, or modulate the fitness of metastatic cells once they establish. Direct evidence remains heterogeneous, but the distinction is experimentally important because preconditioning and post-arrival support make different predictions and require different interventions.

Treatment should be treated as a biologically active stage, but treatment-associated neural changes need to be separated into three mechanisms. First, direct treatment-related nerve or Schwann-cell injury may follow surgery, radiotherapy, neurotoxic chemotherapy, or a drug with direct neural effects. Second, neural architecture may change secondarily as tumor burden falls, inflammation resolves or flares, vessels remodel, and tissue repair proceeds. Third, residual tumor and its neural ecosystem may actively adapt or be selected under therapy. These processes can occur together but are not mechanistically interchangeable. Direct human-tissue evidence in pancreatic ductal adenocarcinoma shows that neoadjuvant chemotherapy is associated with increased nerve density and tertiary lymphoid structure abundance, more mature nerve-proximal tertiary lymphoid structures, and chemotherapy-associated Schwann-cell transcriptional changes [40]. These group comparisons support treatment-associated remodeling, but they do not establish that neural changes cause treatment response or justify denervation as a therapeutic strategy. Serial pre/post-treatment specimens, matched untreated controls, neural-function assays, and immune/stromal measurements are needed to distinguish injury from regression-associated remodeling and active adaptation.

**Figure 1:**
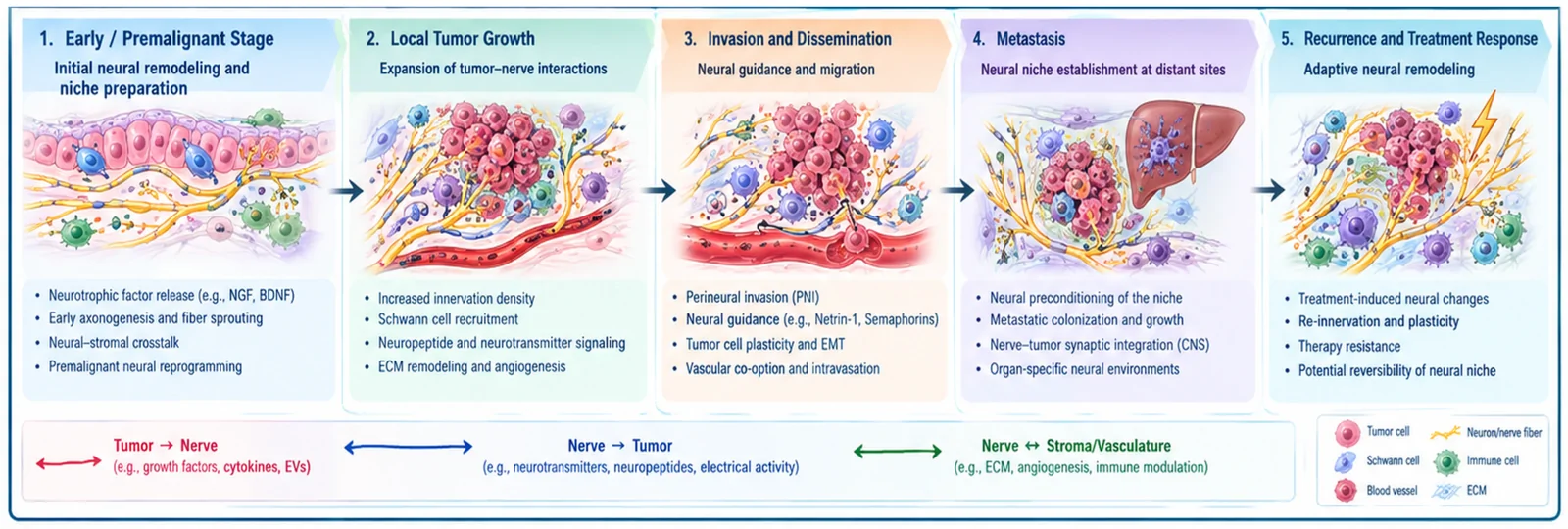
Temporal model of neural remodeling across cancer evolution. Neural change may precede, accompany or follow malignant transitions; the figure distinguishes disease stage from the three separate readouts of neural abundance, neural state and neural activity. This is a conceptual synthesis, not a universal trajectory.

Recurrence therefore deserves explicit inclusion in the neural timeline. Key unresolved questions are whether neural states normalize after complete tumor removal, whether residual nerves retain a “memory” of the tumor, whether therapy selects for neural-adapted residual cells, and whether recurrent tumors rebuild neural networks differently from untreated primaries. These questions are particularly amenable to serial spatial pathology and longitudinal imaging because they cannot be answered reliably from a single static specimen. The temporal stages, representative neural phenotypes, and associated evidentiary requirements are summarized in Table 2 and illustrated conceptually in Figure 1.

## 5. Molecular Mechanisms of Neural Recruitment and Reprogramming

### 5.1. Neurotrophins and Axon-Guidance Programs

Tumors recruit and reprogram neural structures through overlapping classes of trophic and guidance cues rather than through a single canonical pathway. NGF, BDNF, GDNF and related neurotrophins can promote neurite growth or neuronal survival, whereas netrins, semaphorins, ephrins, slits and plexin-family pathways regulate directional growth, adhesion and tissue positioning. Developmental guidance programs are particularly attractive cancer mechanisms because malignant tissues can reactivate signaling systems that are normally restricted to development or tissue repair [3,4,37,39].

The breast cancer literature illustrates a distinct principle: neural signals can change tumor-cell behavior through axon-guidance receptors without necessarily requiring a large increase in total nerve density. Sensory nerves are enriched in human triple-negative breast cancer, and direct neuron-cancer contact can increase tumor-cell attachment and migration through PlexinB3-dependent mechanisms. The important variable is therefore not simply whether nerves are present, but whether tumor cells are competent to recognize and exploit specific axonal cues [16].

Semaphorin signaling further illustrates pathway multiplexing. In pancreatic cancer, SEMA3D promotes disease progression through macrophage reprogramming, showing that an axon-guidance molecule can have a major tumor effect through an intermediary stromal compartment rather than solely through neuronal recruitment. This expands the concept of neural guidance from “neurons attract tumors” to a broader developmental signaling network that can simultaneously organize neural, immune and malignant states [41].

### 5.2. Metabolic and Lipid-Mediated Communication

Neural remodeling is also embedded in tumor metabolism. The recent pancreatic ganglioside study suggests that epithelial lipid remodeling can actively induce neuritogenesis during ADM and PanIN development, identifying glycosphingolipid metabolism as a molecular interface between epithelial state and neural plasticity. This is important because it links neural remodeling to a metabolic phenotype that may be measurable independently of nerve architecture [38].

Neurons can reciprocally influence tumor metabolism. Pancreatic cancer models have shown that neurons can release serine to support protein synthesis in malignant cells, providing an example of a neural metabolic contribution that is not captured by classical neurotransmitter-centered models. Such findings imply that the neural niche can function as a local metabolic resource as well as a signaling compartment [42].

Catecholamines provide another metabolic bridge. In experimental prostate cancer, adrenergic nerves activate vascular and metabolic programs. In hepatocellular carcinoma, human tissue analyses identified abundant sympathetic fibers near the invasion front, while catecholamine exposure induced inflammatory, proliferative, and ECM-remodeling programs in differentiated tumor hepatocytes and primary human hepatocytes. These findings support a mechanistic link between catecholaminergic signaling and the tumor-cell state, but they do not by themselves establish that sympathetic innervation is a clinical driver of human HCC progression [2,23].

### 5.3. Tumor Secretomes, Extracellular Vesicles, and Stromal Feed-Forward Loops

Primary experimental evidence supports a tumor-to-nerve role for extracellular vesicles in axonogenesis. In head and neck cancer, tumor-derived exosomes induced neurite outgrowth in neuronal models, exosome-deficient tumors were less innervated in mice, and exosome-packaged EphrinB1 potentiated axonogenesis. These data establish a mechanism for structural neural recruitment in an experimental setting but do not by themselves prove that extracellular vesicles broadly reprogram neurotransmitter release or neuronal transcription across cancers [43].

Stromal cells increasingly appear to be intermediates rather than bystanders. Fibroblasts can provide the ECM and trophic support to axons, while neural signals can modify fibroblast phenotype and matrix deposition. In TNBC, sensory-neuron-derived CGRP was linked to a dense ECM and immune-excluded state, indicating that neural activity can reshape stromal architecture and thereby alter immune and mechanical properties indirectly (Table 3) [44].

## 6. Neuronal and Glial Identity: From Nerve Class to Disease-Associated Neural States

Peripheral organs contain sympathetic, parasympathetic, sensory, enteric and organ-specific neuronal populations. Their projections, receptor repertoires, neurotransmitters and target tissues differ; consequently, “nerve density” is biologically under-specified unless the neuronal population is known. The prostate literature demonstrated that sympathetic and parasympathetic inputs can have different effects on cancer initiation and dissemination, while gastric models established cholinergic dependence of epithelial growth [1,15].

Projection-aware neuronal profiling is therefore critical. Trace-n-Seq directly addresses the anatomical problem that neuronal somata are often located in distant ganglia rather than within tumor tissue. By retrogradely labeling projections and sequencing the corresponding neuronal cell bodies, this approach can identify the neurons that actually innervate a target organ and can reveal disease-associated transcriptional states that conventional tumor scRNA-seq cannot capture [25].

The next conceptual step is to distinguish lineage from state. A sensory neuron remains a sensory neuron while its transcriptional program, excitability, neuropeptide production, or response to tumor-derived cues changes. In pancreatic cancer, single-neuron analysis demonstrates this possibility at scale. In head and neck cancer, loss of p53 in tumor cells has been linked to acquisition of neuronal features, illustrating that tumor-cell plasticity can complicate the distinction between neural recruitment and tumor-cell reprogramming [25,46].

Glial cells warrant parallel attention. Schwann cells participate in nerve repair and can be co-opted by tumor cells. They can promote dispersion, migration, and invasion through direct contact and NCAM1-dependent mechanisms, while Schwann-cell recruitment has been observed near neoplastic sites before classical invasion. These observations support a broader definition of the neural ecosystem in which Schwann cells are active regulators of tumor movement and tissue remodeling rather than inert components of the nerve sheath [34,35].

This glial perspective also clarifies the relationship between neural remodeling and PNI. PNI is a pathological manifestation of tumor–nerve interaction, but the cellular machinery that permits neural migration may begin before malignant cells have entered the nerve sheath. Future studies should therefore map Schwann-cell states together with axonal architecture and tumor-cell phenotypes to determine whether PNI represents a late endpoint of an already established neural niche or a distinct biological transition.

## 7. Spatial Organization: The Neural Microdomain

Global nerve density is an incomplete phenotype because neural fibers are elongated, branched structures. Two tumors with similar neural density may differ in axonal orientation, branching, terminal density, distance to vessels, proximity to fibroblast-rich regions, and adjacency to distinct tumor-cell states. Fine unmyelinated fibers and terminal structures may also be underestimated by conventional two-dimensional histology [3,4,28,29,30,31,32,33].

We propose the neural microdomain as an operational spatial unit, but do not imply that a universal radius or validated clinical assay already exists. A candidate microdomain should be defined within a prespecified tissue compartment and imaging/sampling window; dimensions must be reported in physical units and selected according to specimen architecture and platform resolution rather than an arbitrary universal distance. At minimum, its record should include four elements: (1) neural identity, established by a validated pan-neural marker and subtype or projection evidence where available; (2) topology, quantified at a stated 2D/3D resolution using measures such as fiber length, branch density, orientation, terminal distribution, or distance to neighboring cells; (3) a molecular neighborhood containing at least one defined tumor-cell state and one adjacent immune, stromal, or vascular compartment; and (4) at least one spatially localized molecular interface, such as a ligand/receptor pair or protein/RNA signal. These criteria define an anatomically and molecularly characterized candidate microdomain, not proof of communication. A claim of functional validation additionally requires direct neural activity/output measurement or a controlled perturbation linked to a phenotype, preferably with an orthogonal test or rescue. Archived human tissue alone will ordinarily support anatomical/molecular characterization, not functional causality.

Reproducibility must be tested rather than assumed. Studies should prespecify sampling boundaries, marker panels, spatial resolution, segmentation rules, distance metrics, and the denominator used for density or topology. At least a subset of fields should be annotated independently and blinded, with interobserver agreement reported using a suitable statistic (for example, an intraclass correlation coefficient for continuous metrics or kappa for categories). Segmentation agreement, within-specimen variability, between-specimen variability, and cross-platform concordance should be quantified, followed by validation in an independent patient cohort. Spatial transcriptomics provides broad molecular context but varies in spot size, sensitivity, and transcriptome coverage. Image-based methods such as MERFISH permit finer localization but require targeted panels and imaging infrastructure; near-cellular platforms improve granularity while retaining trade-offs in coverage and scalability [28,29,30,31,32,33].

For neural biology, multimodal integration is particularly important because axons can extend far from the transcriptional soma. A spatial dataset can identify a tumor or stromal state adjacent to an axonal structure without proving which neuronal population generated it. Conversely, a traced neuronal population can be molecularly defined without revealing the exact cells it contacts in the tumor. The most informative experiments therefore connect neuronal projection identity to local spatial context through coordinated tracing, morphology and spatial molecular profiling [25,27,28,29,30,31,32,33].

**Figure 2:**
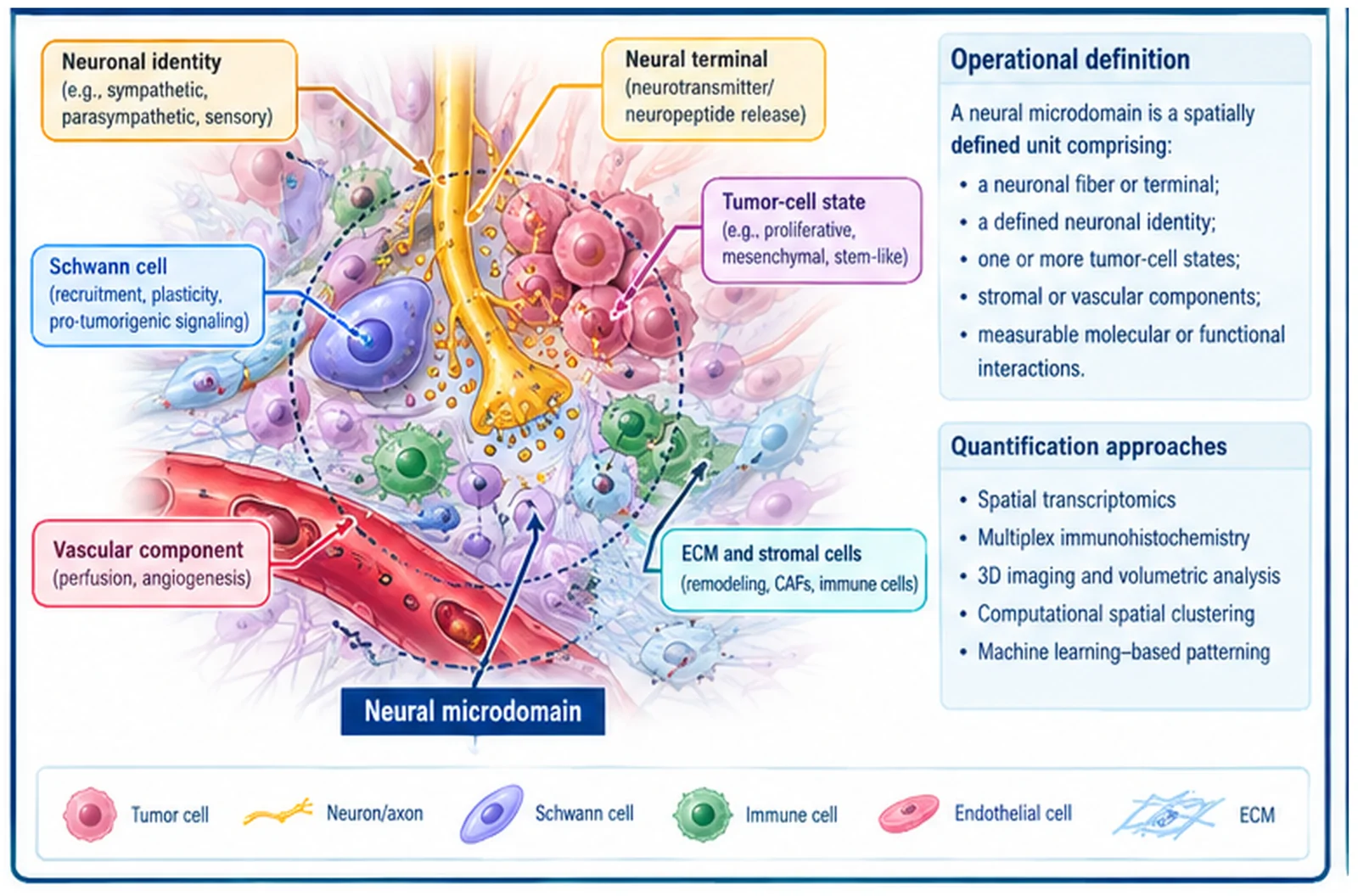
Operational view of a tumor–nerve neural microdomain. A candidate microdomain combines validated neural identity and topology with a mapped tumor-cell state and neighboring immune, stromal or vascular context; functional validation requires direct activity/output measurement or controlled perturbation. The drawing is schematic and not to scale.

Three-dimensional imaging adds network topology that is difficult to infer from serial sections. Tissue clearing and light-sheet or confocal reconstruction can quantify branching, trajectories and proximity to ducts, vessels, and tumor nests. These approaches reduce the risk of mistaking a section through a neighboring nerve bundle for genuine intratumoral innervation, but they introduce processing-dependent changes in tissue dimensions, antibody penetration, segmentation, and signal detection. Such measurements should therefore be normalized against appropriate anatomical references and, whenever possible, validated by orthogonal histology (Figure 2).

## 8. Functional Consequences for Tumor-Cell State and Tissue Behavior

The strongest causal evidence for neural regulation arises when a defined neural component is perturbed and the intervention changes a measurable tumor phenotype while alternative systemic explanations are addressed. Neural inputs can influence proliferation, migration, angiogenesis, cell-state plasticity, metabolism, ECM organization and, in selected settings, direct electrical activity [1,2,3,15,16,17,18,19].

### 8.1. Proliferation, Plasticity, and Stem-like States

Autonomic signaling can act directly on epithelial or stromal compartments. Prostate studies demonstrated branch-specific effects on tumor development and dissemination, whereas gastric denervation reduced tumor growth through effects involving muscarinic signaling and Wnt-dependent stem-cell programs. The gastric model is especially informative because denervation was accompanied by reduced tumor incidence and progression and because organoid experiments linked neuronal input to epithelial growth [1,15,45].

Pancreatic models show why pathway effects must be interpreted by stage and cellular target. Netrin-1/DCC-associated sympathetic remodeling can restrain progression in precursor lesions, whereas Netrin-1/NEO1 signaling supports growth, the epithelial–mesenchymal transition and stem-like programs in established PDAC [37,39]. The mechanistic evidence is summarized in Section 5.1; here, the key functional lesson is that one ligand cannot be assigned a universal pro- or antitumor role without specifying disease stage, receptor-bearing cell, and measured endpoint.

Neural cues can also alter tumor-cell migration without increasing bulk growth. In TNBC, sensory nerves enhance adhesion and migration through PlexinB3, suggesting that neural interfaces can selectively increase dissemination competence. This is conceptually important because a neural phenotype may change the probability of invasion or metastasis without producing a proportional change in primary tumor volume [16].

### 8.2. Angiogenesis, ECM, and Metabolic Adaptation

Neural regulation extends beyond direct tumor-cell signaling. Adrenergic nerves can activate vascular and metabolic programs, illustrating how neural input can organize the physical conditions needed for tumor expansion. In TNBC, sensory-neuron-derived CGRP can alter fibroblast phenotype and collagen deposition, producing a dense ECM associated with immune exclusion [2,44].

The ECM should therefore be viewed as a major interface between neural and malignant compartments. Matrix stiffness, collagen organization and interstitial architecture can affect cell migration, immune-cell access and drug penetration. A neural intervention that changes the ECM may consequently have effects that are not visible in tumor-cell signaling assays alone and may interact with therapies whose efficacy depends on tissue penetration or immune access.

### 8.3. Neural Signaling and the Tumor Immune Microenvironment

Neural influences on antitumor immunity are not uniformly suppressive or stimulatory. Their direction depends on neuronal branch, receptor expression, immune-cell target, tissue context and disease stage. In TNBC models, sensory-neuron-derived CGRP is linked to fibroblast activation, a dense extracellular matrix and immune exclusion; the mechanistic perturbation evidence is preclinical, whereas the presence of corresponding neural features in human tumors is observational [44]. In colorectal cancer, human tissue analyses have reported local sympathetic degeneration, while experimental work indicates that norepinephrine acting through α2-adrenergic signaling can increase chemokine-associated CD8+ T-cell recruitment and restrain progression in an immune-competent model [21]. This result contrasts with studies of broader chemical denervation and tumor-volume endpoints, discussed in Section 13.2. Other work has identified a sensory–sympathetic tumor–brain axis associated with immune suppression in lung adenocarcinoma models; this is an emerging preclinical circuit, not evidence that the same causal pathway controls human lung cancer immunity [47]. Accordingly, a neural immune phenotype should be reported with the responsible neural population, immune compartment, assay, disease stage and evidence level. Human spatial association, transcriptional co-occurrence and preclinical perturbation should be described separately; no universal “neural immunosuppression” mechanism can be inferred across cancers.

### 8.4. Electrical and Synaptic Signaling

CNS tumors provide the clearest demonstration that neural signaling can be electrical rather than primarily chemical. Glioma cells can receive glutamatergic synaptic input, depolarize, and participate in electrically coupled networks. Human iPSC-derived co-culture models have demonstrated cholinergic neuron-to-glioblastoma synaptic interactions, providing an experimentally tractable human platform [17,18,26].

The biological boundary is now becoming less absolute. The 2025 SCLC study found that pulmonary innervation contributes to primary tumor initiation and development in experimental models, while analyses of human brain metastases and functional neuronal co-culture models showed that neuronal activity can promote intracranial SCLC growth. This illustrates a single cancer type engaging distinct neural mechanisms by anatomical site and reinforces the need to distinguish peripheral neural signaling from direct CNS circuit integration [19].

## 9. Dissemination, Metastasis, and Systemic Neural Circuit Co-Option

Neural regulation of dissemination can be conceptualized at four levels: local tumor-cell migration, access to vascular or lymphatic routes, preconditioning of distant tissues, and post-arrival adaptation within the metastatic site. These levels should not be conflated because the same neural signal may influence one stage without controlling another.

At the primary site, nerve fibers can provide directional or adhesive interfaces that promote tumor-cell movement. This mechanism differs from PNI because the relevant phenomenon may occur before malignant cells invade the nerve sheath. At metastatic sites, the pre-existing neural architecture of the target organ provides a different environment, and disseminated cells may encounter organ-specific neuronal, glial and stromal states.

An important emerging example is colorectal cancer, in which a polysynaptic brain–gut circuit linking the lateral septum and enteric cholinergic neurons sustained tumor growth in mice, and chronic stress potentiated the circuit. This study demonstrates a testable systemic mechanism in a preclinical model; its clinical relevance and generalizability to human colorectal cancer remain uncertain [22]. The recently described lung tumor–brain–sympathetic axis likewise expands the possible circuit scale but is supported by experimental mechanistic evidence rather than clinical causal data [47]. These results should motivate—not substitute for—human validation and do not establish that systemic neural circuits determine metastatic organotropism.

**Figure 3:**
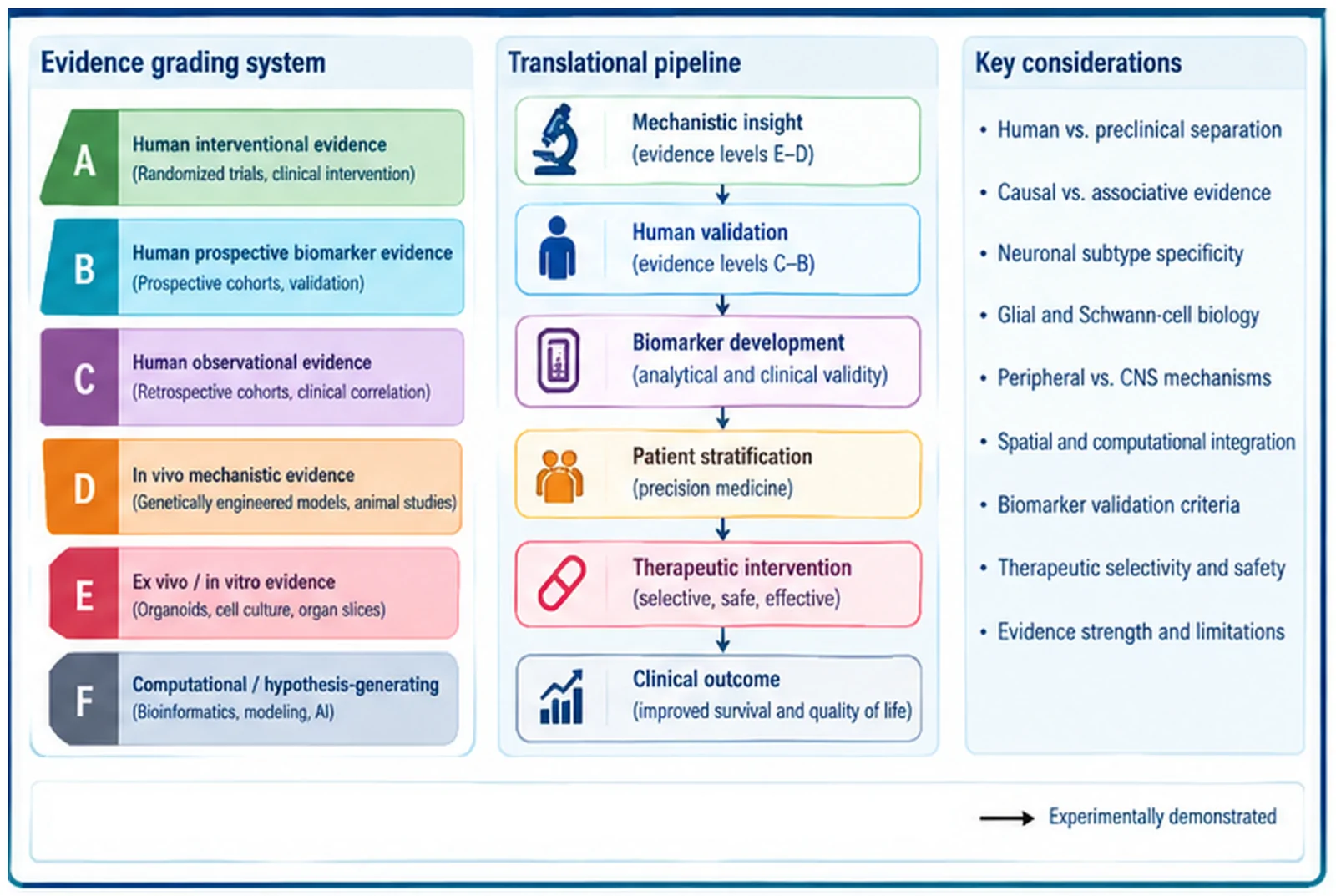
Evidence calibration from anatomical localization to clinical intervention. Each evidence domain supports a different claim; molecular or spatial detail does not, by itself, establish causality or clinical utility.

Metastatic organotropism remains unresolved. A rigorous test would require matched primary and metastatic lesions, projection-specific tracing, lineage barcoding, spatial profiling, and longitudinal perturbation. Studies should distinguish neural preconditioning of a target organ before tumor-cell arrival from local neural support after colonization; endpoint data from established metastases cannot discriminate these possibilities. Current claims of neural control of organotropism should therefore be labeled emerging or preclinical unless pre-arrival timing and causal dependence have been directly demonstrated (Table 4 and Figure 3).

## 10. Experimental Toolkit and Evidence Hierarchy for Causal Inference

Histopathology and multiplex immunofluorescence remain the anatomical foundation. Broad neuronal markers can identify nerve fibers, while subtype markers such as tyrosine hydroxylase, choline acetyltransferase, vesicular acetylcholine transporter and sensory-neuropeptide markers provide lineage information. However, marker positivity alone does not establish functional activity and can be influenced by tissue injury, inflammation, treatment, or altered differentiation [4,32].

Three-dimensional imaging adds network geometry and can distinguish true intratumoral fibers from adjacent bundles. Retrograde tracing answers a different question: which neuronal cell bodies project into the target tissue? Trace-n-Seq links these approaches to single-cell transcriptomics and is particularly valuable because distal neuronal somata are systematically missed by conventional tumor sequencing. Newer transsynaptic tracing approaches can interrogate circuit connectivity in glioblastoma, although these methods remain primarily preclinical and require careful control of tracer specificity and experimental perturbation [25,27].

Single-cell and single-nucleus sequencing resolve cellular states but should be interpreted in anatomical context. In extracranial tumors, neuronal somata often lie in distant ganglia; a tumor scRNA-seq dataset can therefore underestimate the neural compartment even when neural processes are abundant. Conversely, neuronal transcriptomes may not represent the local axonal output reaching the tumor. Paired ganglion-tumor sampling or projection-specific labeling is therefore preferable when neuronal molecular identity is a principal endpoint [25,28,29,30,31,32,33].

Functional assays provide the bridge from anatomy to mechanism. Dorsal root ganglion cultures, whole-mount co-cultures, organoids and microfluidic systems can control distance and contact between neurons and cancer cells. Electrophysiology, calcium imaging, optogenetics and chemogenetics can manipulate activity with greater subtype or temporal specificity than global denervation. Genetic deletion and receptor-selective pharmacology can then test whether an identified pathway is required for the phenotype [17,18,26,27].

**Figure 4:**
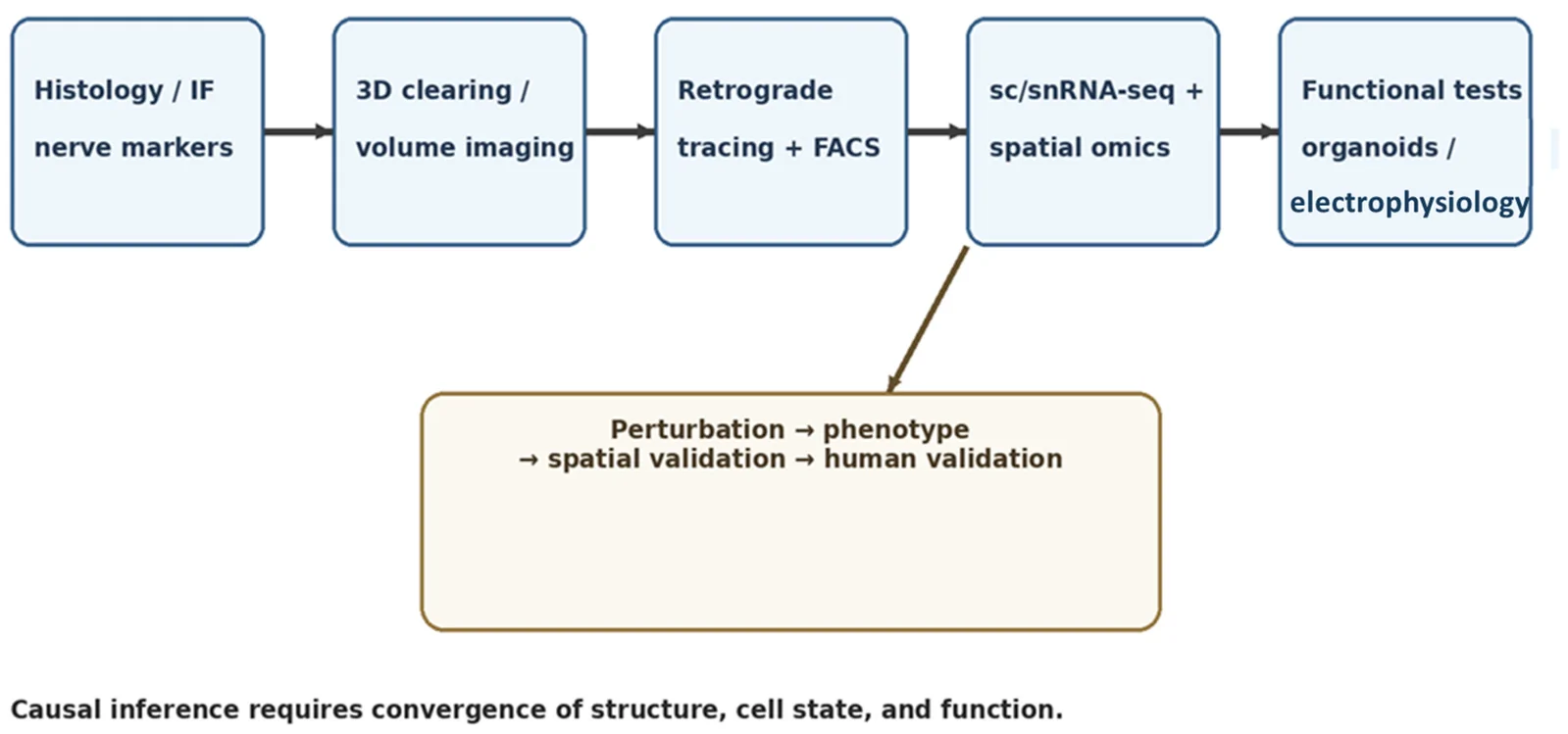
Multiscale workflow for causal dissection of tumor–neural interactions. The workflow links anatomy, projection identity, local cell states, functional assays, orthogonal perturbation/rescue and independent validation, with patient-level inference and confounder control as design guardrails.

No single perturbation is definitive. Surgical and chemical denervation can induce injury responses, while receptor antagonists may affect non-neural cells, genetic deletion may produce developmental compensation, and optogenetic or chemogenetic tools may impose nonphysiological activity patterns. Denervation studies should include sham-operated or procedure-matched controls, confirmation that the intended neural input was functionally interrupted, and prespecified measurements of local inflammation, vascular changes, tissue injury and repair, stress/pain behavior, organ physiology and tumor burden. Where feasible, compare a broad denervation procedure with a local, branch- or subtype-selective perturbation; use an independent orthogonal approach and rescue to test whether the phenotype follows loss of the neural signal rather than the procedure. Receptor-selective interventions should be checked for expression in tumor, immune, vascular and stromal cells. These controls are particularly important when treatment effects could be mediated by appetite, gut motility, blood pressure, wound repair or systemic stress rather than by a tumor-local neural circuit (Table 5 and Figure 4).

## 11. Computational Integration and Quantitative Spatial Analysis

Cancer neuroscience increasingly generates datasets spanning histology, volumetric imaging, neural tracing, single-cell or single-nucleus sequencing, spatial transcriptomics, multiplexed imaging, proteomics, and functional measurements. The principal computational challenge is not merely integration of datasets but correct attribution of a spatial or molecular association to the appropriate biological compartment while controlling for tissue composition, technical batch effects, spatial autocorrelation and reference mismatch [32,33].

Cell-type deconvolution should be interpreted cautiously when the neuronal reference atlas is derived from healthy ganglia. Disease-associated neurons may exhibit states that are absent from the reference, producing misclassification or underestimation. Projection-specific neuronal datasets therefore offer a biologically more appropriate reference where available [25].

Ligand-receptor analysis is hypothesis-generating rather than proof of communication. Co-expression without spatial proximity, receptor localization, ligand availability, or downstream pathway activation should not be described as functional communication. The evidentiary threshold should rise from computational prediction to spatial localization, then to perturbation and rescue. The same principle applies to inferred cell–cell communication in spatial transcriptomic datasets [32,33].

Image analysis should move beyond total nerve density toward network descriptors such as total fiber length, branch density, orientation, tortuosity, terminal density, distance to defined cellular states, and graph connectivity. The patient—not each cell, field, nerve segment or region of interest—must remain the primary inferential unit for clinical associations. Measurements from multiple fields or cells are nested within the same patient and should be aggregated using prespecified patient-level summaries or modeled with hierarchical/mixed-effects methods that account for clustering and spatial autocorrelation. Sample-size planning should be based on the number of independent patients, not the number of cells captured. Batch effects, multiple testing, segmentation uncertainty and missingness should be reported, and the candidate metrics should be tested in an independent cohort. Graph-based representations can capture the connected nature of fibers, but their associations with outcome still require patient-level inference and external validation.

Longitudinal analysis offers a particularly important opportunity. Static comparisons among normal tissue, premalignancy, primary tumor, and metastasis cannot reliably establish directionality. Aligning neural and epithelial trajectories across time, or across experimentally staged disease, can generate testable hypotheses about whether neural changes precede, accompany, or follow transitions in tumor and stromal state; causal attribution still requires longitudinal perturbation and appropriate controls.

## 12. Translational Opportunities, Biomarkers, and Clinical Constraints

### 12.1. Neural Biomarkers

Potential neural biomarkers include histological nerve density, subtype-specific neural markers, spatial nerve–tumor relationships, neuronal or glial transcriptional signatures, circulating neural factors, and functional imaging. Three uses must be distinguished. A prognostic biomarker identifies outcome risk under a defined clinical context irrespective of a particular treatment effect. A predictive biomarker identifies variation in the benefit or harm of a specified treatment and ideally demonstrates a treatment-by-biomarker interaction. A response or pharmacodynamic biomarker changes after exposure and may indicate biological target engagement or tumor response, but does not alone establish long-term benefit. A feature can be prognostic without being predictive, and a treatment-associated change is not automatically either.

A clinically useful neural biomarker must show analytical validity (the assay measures the intended feature reproducibly), biological validity (the feature reflects a plausible neural process), clinical validity (the association or interaction replicates in the intended population), and clinical utility (using the result improves a defined decision or outcome). Pathology-based assays require standardized tissue selection, staining, segmentation, reference regions, thresholds and independent external validation. Recent PDAC nerve-proximal tertiary lymphoid structure and TNBC innervation-signature findings are promising human observational or retrospective candidates; they should not be presented as validated clinical tests until prespecified thresholds and intended uses are independently confirmed [40,48].

### 12.2. Therapeutic Strategies

Therapeutic strategies under investigation include adrenergic or muscarinic modulation, neurotrophin and axon-guidance pathway inhibition, sensory-neuropeptide blockade, targeted denervation, and activity-selective neuromodulation. These approaches are not equivalent: their targets, causal evidence, anatomical reach and toxicities differ. At present, routine pathology/IHC, digital nerve quantification, and selected spatial assays are technically feasible for exploratory phenotyping; neuroactive-drug repurposing is feasible only within carefully designed prospective trials. Circuit-selective, spatially restricted neuromodulation and patient-specific projection tracing remain investigational or longer-term approaches [49,50].

Mechanistically selective approaches may offer a narrower therapeutic window than broad neuroablation when a specific disease-associated circuit has been identified. However, receptor-directed therapies may fail when several neural populations converge on the same tumor state, while global denervation can disrupt blood-pressure regulation, gastrointestinal motility, sensory function, glandular secretion, tissue repair, or other essential physiology. The therapeutic strategy must therefore be matched to tumor-localized receptor expression, neuronal subtype, anatomical accessibility and disease stage.

Drug repurposing is attractive because beta-blockers and other neuromodulators are pharmacologically tractable, but retrospective associations remain vulnerable to indication bias, comorbidity, dose heterogeneity, treatment selection and immortal-time bias. In a phase II randomized perioperative breast-cancer study, propranolol altered metastasis-related biomarkers; the study did not establish improved recurrence or survival and should not be interpreted as evidence for routine anticancer use [51]. Prospective trials should define whether the intended endpoint is target engagement, a biomarker of response, pathologic response, recurrence, or survival, and should prespecify safety monitoring and patient selection.

Combination therapy provides a rational route only when the neural mechanism predicts a complementary therapeutic effect. If neural signaling primarily remodels the ECM, combinations should be selected according to tissue penetration or immune exclusion; if the dominant effect is tumor-cell receptor signaling, pathway-matched targeted therapy may be more logical. Empirical stacking of neuromodulators with standard cancer drugs without mechanistic justification is unlikely to provide a coherent translational framework.

Patient selection will probably be essential. Tumor lineage, genotype, neural subtype, stage, treatment exposure, autonomic state, immune context and comorbid physiology can change the direction and magnitude of neural effects. The conflicting colorectal results and tumor-restraining sympathetic effects reported in melanoma demonstrate why the choice of target must follow a demonstrated disease-specific dependency rather than nerve presence alone [20,21,24].

### 12.3. Clinical Translation and Safety

Clinical translation must preserve the distinction between pathological neural circuits and indispensable physiological neural functions. Current translationally feasible steps include standardized pathology-based phenotyping, digital image analysis, spatial profiling in selected centers, and prospective biomarker studies. By contrast, local nerve ablation, circuit-selective interventions, and spatially restricted neuromodulation remain investigational; their clinical feasibility, reversibility and therapeutic window have not been established broadly. Early trials should include cardiovascular, gastrointestinal, sensory, pain, wound-healing and tissue-repair outcomes, as well as evidence that the proposed neural target was engaged in the tumor. Pancreatic denervation strategies should be described as preclinical and model-dependent unless supported by interventional human evidence (Table 6).

## 13. Evidence Limitations, Causal Inference and Translational Readiness

### 13.1. Measurement Limitations and Reproducibility

Neural elements are elongated, spatially sparse and often sampled indirectly because neuronal somata may reside in distant ganglia. Tumor scRNA-seq or snRNA-seq may therefore underestimate the neural compartment, while routine two-dimensional histology can miss fine axons and terminals. Spatial transcriptomic platforms differ in capture geometry, sensitivity and resolution; computational reference atlases may omit disease-associated neuronal states. Species, strain, organ architecture, tumor model, immune competence, tissue processing and marker specificity further constrain cross-study comparison [25,27,28,29,30,31,32,33].

The neural microdomain remains a research construct rather than a standardized diagnostic assay. To support reproducibility, studies should report the tissue compartment, sampling window, imaging resolution, validated neural and subtype markers, segmentation method, topology/distance metrics, neighboring cell states and the molecular interface measured. A prespecified annotation protocol should be applied blinded to clinical outcome, with independent reviewers, interobserver agreement, segmentation reproducibility and within-/between-specimen variation reported. Platform concordance and independent patient-cohort validation are necessary before a proposed microdomain score can be treated as transportable. A single universal radius is not justified; distances must be reported in physical units and interpreted in relation to tissue architecture and assay resolution.

Computational analyses should treat patients as the primary inferential unit. Multiple cells, fields, nerve segments or spatial spots from one specimen are nested observations, not independent patients. Prespecified patient-level summaries or mixed-effects models should account for clustering and spatial autocorrelation, and sample-size planning should reflect the number of independent patients. Batch effects, multiple testing, missingness and segmentation uncertainty should be disclosed. These requirements apply equally to nerve density, graph-based metrics, spatial proximity and candidate neural signatures.

### 13.2. Causal Inference Limitations and Directionality Disputes

Denervation is not a biologically neutral subtraction of neural input. Surgical or chemical nerve interruption can trigger local injury, inflammation, vascular changes, tissue repair and systemic physiological effects; broad interventions can also affect multiple neural branches. Pharmacological antagonists may act on tumor, immune, vascular or stromal receptors, and genetic or activity-selective tools may have compensatory or nonphysiological effects. Strong designs should include sham/procedure-matched controls, validation of effective functional denervation, local and subtype-selective approaches where feasible, orthogonal perturbations, rescue, temporal controls and measures of inflammation, vascularity, stress/pain, organ function, tissue repair and tumor phenotype. These controls help distinguish a neural mechanism from effects attributable to the procedure, drug off-target activity or altered whole-organ physiology.

Colorectal cancer illustrates the need to compare designs and endpoints directly rather than resolving disagreement with generic appeals to context. Wang et al. associated higher nerve density and perineural invasion with poorer outcome in a human cohort and reported smaller tumors after broad chemical denervation in a murine rectal-cancer model. That design combined observational human measures with a perturbation strategy that affected multiple neural branches, and the key experimental endpoint was tumor burden [20]. Chen et al. instead emphasized local sympathetic degeneration in human tissue and experimental models; sympathetic ablation worsened progression in an AOM/DSS model, while norepinephrine/α2-adrenergic signaling was linked to chemokine production and CD8+ T-cell recruitment [21]. The studies therefore differ in neural branch specificity, perturbation breadth, immune mechanism, disease model and endpoint. Their results are not resolved by labeling all sympathetic signaling protective or all innervation tumor-promoting. Direct reconciliation requires matched models, branch-specific interventions, immune-competent controls, comparable endpoints, functional verification and rescue. The systemic brain–gut circuit is an additional preclinical mechanism, not a substitute for that comparison [22].

More generally, anatomical proximity, ligand–receptor co-expression and human outcome associations are not equivalent to demonstrated signaling. A causal claim should be reserved for experiments that perturb a defined neural component, measure a relevant phenotype, control plausible alternatives, and ideally reproduce or rescue the effect in an independent model. When such evidence is absent, findings should be labeled anatomical, molecular, hypothesis-generating or human observational, as appropriate (Table 7).

### 13.3. Translational Limitations, Biomarkers and Safety

Evidence involving treatment-associated neural remodeling, metastatic organotropism, systemic circuit co-option and neural biomarkers remains uneven and should be described as emerging or preclinical at the point of first mention unless a prospective human study establishes the stated claim. In a PDAC series comparing 86 treatment-naïve with 49 neoadjuvant-treated specimens, nerve-proximal tertiary lymphoid structures and Schwann-cell states were associated with treatment response; this is valuable human observational evidence, not proof that nerves cause chemotherapy sensitivity [40]. A multi-cohort TNBC report proposed a TLR7-associated innervation signature with outcome and treatment-response associations, but this remains a candidate signature requiring independent, prospective validation [48]. The reported sensory–sympathetic tumor–brain axis and GDF15-associated CCN3-positive Schwann-cell state provide mechanistic leads from experimental models; they should not be generalized to all lung tumors, all Schwann cells, or human therapeutic settings without further evidence [47,54].

Neural biomarkers should be qualified for a defined intended use. A prognostic marker relates to outcome in a specified setting; a predictive marker identifies differential benefit from a treatment, ideally through a treatment-by-marker interaction; and a response/pharmacodynamic marker tracks change after treatment without necessarily demonstrating durable clinical benefit. Candidate nerve density, spatial neural features, nerve-proximal tertiary lymphoid structures and neural transcriptional signatures require analytical standardization, prespecified cut-offs, independent validation, incremental-value testing and prospective evaluation against a defined decision. Association with outcome alone does not establish predictive value or clinical utility.

**Figure 5:**
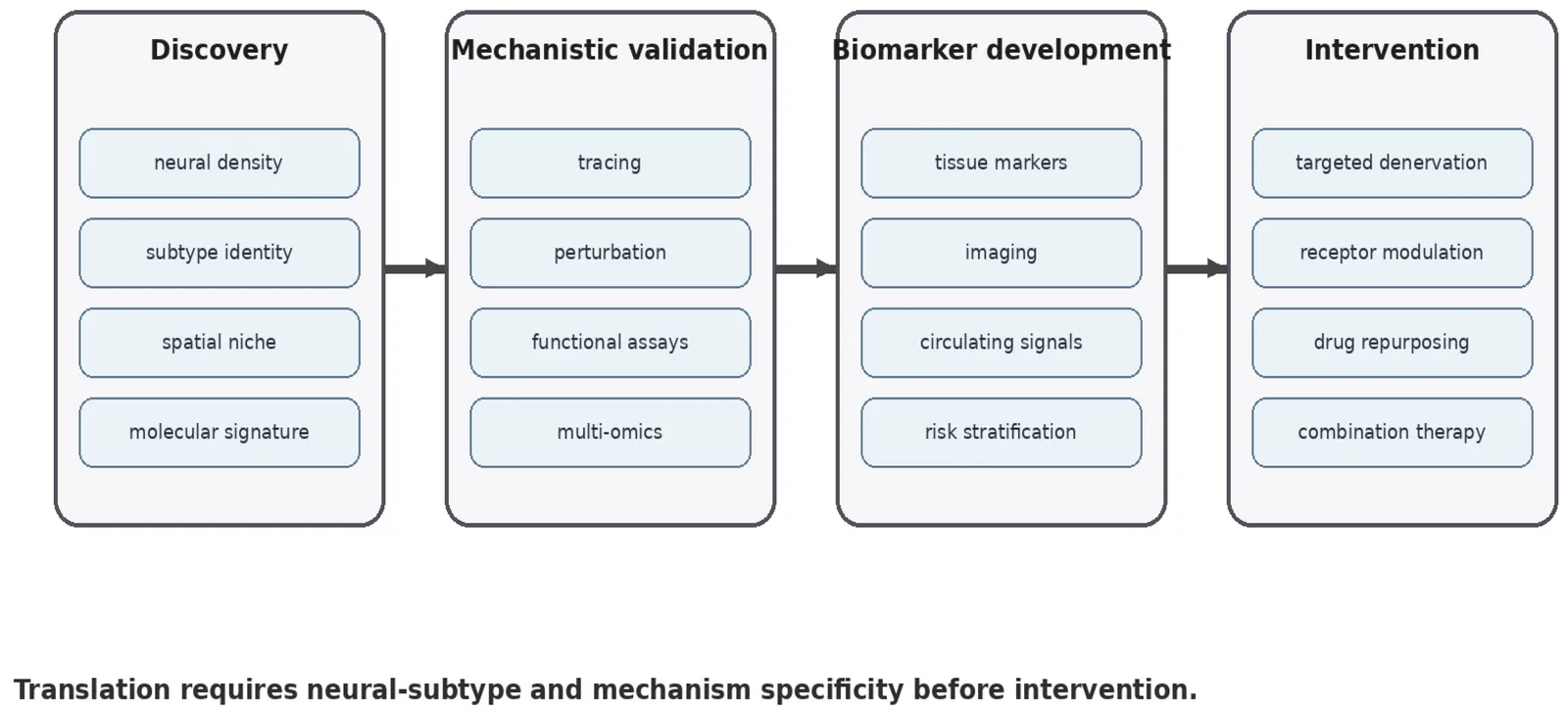
Translational pathway from neural phenotyping to clinical decision-making. Pathology-based phenotyping and external validation are feasible near-term priorities, whereas circuit-selective or spatially restricted interventions remain investigational. A phase II propranolol study altered breast-cancer biomarkers but did not establish recurrence or survival benefit.

For example, a phase II randomized study of perioperative propranolol in breast cancer demonstrated modulation of metastasis-related biomarkers, but did not establish improved recurrence or survival and should not support routine anticancer use [51]. Pancreatic denervation and focal neuromodulation remain preclinical or investigational unless supported by interventional human evidence. Near-term feasible priorities include standardized pathology-based phenotyping, digital image analysis, prospective observational validation and carefully designed biomarker-driven trials. More distant possibilities include projection-aware patient-specific circuit mapping and spatially restricted neuromodulation; both require proof of tumor target engagement, reversibility and a favorable safety margin. Trial design should monitor cardiovascular, gastrointestinal, sensory, pain, wound-healing and tissue-repair effects and should preserve essential neural physiology [49,50,55,56] (Figure 5, Table 8 and Table 9).

## 14. Priority Questions for the Next Phase of Cancer Neuroscience

Priority questions are: (1) which neural changes arise earliest in each organ and are reproducibly detectable in human precursor lesions; (2) which tumor or stromal signals select particular neuronal subtypes and whether these states are stable or reversible; (3) whether a neural microdomain can be measured reproducibly and add information beyond conventional pathology; (4) which effects are mediated directly by tumor-cell receptors versus stromal, vascular, metabolic or immune intermediates; (5) how treatment alters neural architecture and whether those changes reflect injury, regression-associated repair or active adaptation; (6) whether candidate neural biomarkers are prognostic, predictive or pharmacodynamic; and (7) whether neural interventions can be sufficiently selective to preserve essential organ physiology. These questions require prespecified patient-level endpoints, longitudinal sampling where feasible, and explicit separation of human observations from preclinical mechanisms.

A standardized experimental sequence should define the anatomical phenotype, identify the projection source, resolve neuronal and glial states, map neighboring tumor and stromal microdomains, quantify abundance/state/activity separately, perturb the candidate circuit with orthogonal approaches, test rescue, and validate the phenotype in an independent model and human cohort where possible. Clinical datasets should use the patient as the primary inferential unit and report reproducibility, confounding and missingness transparently. This sequence will reduce overinterpretation of descriptive datasets and accelerate translation of mechanisms that are both causal and measurable.

Contemporary single-cell and spatial studies, together with recent conceptual reviews, reinforce the need to interpret neural remodeling through spatial context, cellular identity, disease stage and functional evidence rather than nerve abundance alone [25,44,52,53,54,55,56,57,58].

## 15. Conclusions

Neural remodeling is a dynamic component of cancer evolution, not a single late-stage anatomical phenomenon. Evidence supports neural changes during precursor remodeling, established disease, invasion, and selected metastatic or treatment-associated states, but the direction of these effects is context-dependent. The pancreatic, colorectal and melanoma examples show why neuronal branch, receptor-bearing cell, disease stage and immune context must be specified before a pathway is regarded as tumor-promoting or tumor-restraining.

The field is moving beyond nerve counts toward integrated assessment of neural abundance, neural state and neural activity, combined with topology and the tumor/immune/stromal neighborhood. A reportable neural microdomain should include validated neural identity, quantitative topology, defined neighboring cell states and a localized molecular interface; functional claims require direct output measurement or controlled perturbation. Histology, projection tracing, spatial assays, electrophysiology and selective perturbation are complementary rather than interchangeable sources of evidence.

The translational objective is not indiscriminate neural blockade. It is to identify stage-specific, subtype-specific and spatially localized dependencies, validate their measurement and causal relevance, and determine whether use of a neural biomarker improves a defined clinical decision. Prognostic associations, treatment-response signals, predictive treatment interactions and evidence of therapeutic benefit must remain distinct. Neural interventions should proceed only with appropriate target engagement and safety safeguards, preserving essential cardiovascular, gastrointestinal, sensory and tissue-repair functions.

## Figures and Tables

**Table 1:** Comparison with contemporary cancer-neuroscience reviews and the distinct operational contribution of this review.

Review/Year	Main Scope	What It Contributes	How the Present Review Complements It
Gillespie and Monje, 2020 [3]	Neural regulation of cancer	Foundational overview of neural influences on cancer biology.	Adds explicit stage-by-stage and evidence-calibrated operational criteria.
Ayala, 2023 [4]	Neuroepithelial interactions	Pathology-oriented view of epithelial–neural relationships.	Adds measurable spatial microdomains and abundance/state/activity separation.
Wang et al., 2025 [5]	Tumor-infiltrating nerves	Survey of nerve recruitment, progression and therapies.	Adds minimum measurement criteria, patient-level statistics and readiness thresholds.
Gu et al., 2026 [6]	Neural regulation and systemic circuit co-option	Integrates chemical, structural and electrical communication.	Adds claim-level evidence labels and cross-stage comparison of directionality.
Li et al., 2026 [7]	Synaptic integration and neuroimmune regulation	Connects neural signaling, CNS integration and immune effects.	Separates local abundance, molecular state, electrical/chemical output and clinical maturity.
Aysit et al., 2026 [8]	Neural niche and neuroimmune crosstalk	Defines neural niches across tumor, neuronal and immune interactions.	Operationalizes a reportable microdomain, with reproducibility and causal criteria.
Magnon and Hondermarck, 2023 [9]	Neural addiction of cancer	Frames cancer dependence on nervous-system signals.	Tests the “dependence” claim against stage, spatial phenotype and perturbation evidence.
Hanahan and Monje, 2023 [10]	Cancer hallmarks and neuroscience	Maps neuroscience onto hallmarks and tumor-microenvironment biology.	Specifies which neural phenotype and evidence domain support each proposed link.
Shi et al., 2022 [11]	Therapeutic opportunities	Reviews translational opportunities and clinical barriers.	Distinguishes feasible assays from investigational circuit-selective approaches.
Liu et al., 2025 [12]	Neural–tumor crosstalk and targeting	Synthesizes reprogramming mechanisms and therapeutic dimensions.	Adds reproducible spatial endpoints and stricter human-versus-preclinical labeling.
Wang et al., 2026 [13]	Concepts and emerging therapy strategies	Extends the discussion to PNI as a distinct route, systemic mechanisms and the neuro–microbic–oncology dimension.	Clarifies that PNI is a pathological route/interface, not a synonym for innervation, and grades clinical readiness.
Jin et al., 2026 [14]	“Neural hijack” and neuroactive-drug repurposing	Discusses neuroactive drugs as potential anticancer interventions.	Adds patient-level biomarker qualification, physiological safety and trial-evidence requirements.
Present review	Temporal, spatial, cellular and functional principles	Integrates time, neural identity, microdomain, activity and directionality.	Provides a measurable microdomain definition, separate abundance/state/activity readouts, evidence-coded synthesis and translational decision criteria.

**Table 2:** Temporal stages of tumor-associated neural remodeling and the evidence required at each stage.

Stage	Representative Neural Phenotype	Core Biological Question	Preferred Evidence	Major Interpretive Risk
Normal tissue	Baseline architecture and neuronal subtypes	What is the tissue reference?	3D mapping, multiplex IF, neuronal atlases	Using an inappropriate anatomical reference
Metaplastic/premalignant	Focal sprouting, guidance signaling, glial recruitment	Does neural change precede invasion?	Longitudinal models, pre-neoplastic human tissue, tracing	Assuming all early remodeling is tumor-promoting
Established primary tumor	Redistributed fibers, subtype shifts, neuronal reprogramming	Which neurons interact with which tumor states?	Tracing, single-cell profiling, spatial omics, volumetric imaging	Confusing density with function
Local invasion/PNI	Neural tracking, Schwann-cell activation, nerve-associated migration	Is the nerve a route, a niche, or both?	Pathology, high-resolution imaging, functional co-culture	Equating PNI with general innervation
Metastatic disease	Target-organ neural adaptation or circuit co-option	Does neural biology affect organotropism or post-arrival fitness?	Matched primary/metastatic tracing, organoids, lineage models	Inferring preconditioning from endpoint data
Treatment/recurrence	Degeneration, persistence, re-innervation, altered neural state	Is neural remodeling reversible and actionable?	Serial tissue, imaging, functional perturbation	Assuming treatment-associated nerve loss is uniformly beneficial

**Table 3:** Major signal classes implicated in tumor-associated neural remodeling, with evidence maturity qualified by context.

Signal Class	Representative Examples	Principal Biological Role	Illustrative Cancer Contexts	Evidence Maturity
Neurotrophins	NGF, BDNF, GDNF [3,4,45]	Neurite growth, survival, reprogramming	Gastric, pancreatic, head and neck	Strong preclinical; variable human validation
Axon-guidance pathways	Netrin-1/DCC or NEO1; semaphorin/plexin; slits/ephrins [16,37,39,41]	Directional growth, adhesion, cell-state signaling	Pancreatic, breast, multiple epithelial tumors	Mechanistic preclinical; stage-dependent
Neuropeptides	CGRP and other sensory neuropeptides [3,4,44]	Local signaling to tumor and stromal cells	Breast, sensory-neuron models	Emerging mechanistic evidence
Catecholaminergic signaling	Norepinephrine, epinephrine, adrenergic receptors [1,2,20,21,23,24]	Growth, angiogenesis, metabolism, immune/stromal regulation	Prostate, pancreas, colorectal, HCC	Heterogeneous and context dependent
Cholinergic signaling	Acetylcholine, muscarinic receptors [15,22,45]	Epithelial growth, stem-like programs, circuit regulation	Gastric, prostate, colorectal	Strong experimental evidence
Lipid/metabolic cues	Gangliosides, serine, metabolites [38,42]	Neural recruitment or tumor metabolic support	Pancreas	Rapidly emerging
Stromal feed-forward signals	CAF-derived trophic/guidance factors, ECM [44]	Couples neural architecture to fibroblast and vascular states	Breast	Emerging; spatially dependent
Extracellular vesicles	Tumor-derived exosomes; EphrinB1 cargo [43]	Neurite outgrowth and tumor innervation	Head and neck cancer; experimental models	Direct mechanistic preclinical evidence; clinical validation limited

**Table 4:** Cross-cancer evidence matrix classified by evidentiary domain and clinical maturity.

Cancer Context	Neural Interface/Principal Finding	Evidence Domains Supported	Direction and Interpretive Limit	Clinical Maturity
Prostate	Autonomic branches differ in effects on initiation and dissemination; adrenergic signaling affects vascular/metabolic programs [1,2].	A, M, PF, HO	Branch- and stage-dependent; density alone is not causal.	Mechanistic preclinical evidence; no validated neural-directed standard.
Gastric	Cholinergic input can support epithelial growth through muscarinic/Wnt programs [15,45].	A, M, PF, HO	Experimental support is strong in tested models; broader generalization needs care.	No established neural-targeted anticancer standard.
Pancreatic precursor/established PDAC	Precursor Netrin-1/DCC remodeling may be protective; Netrin-1/NEO1 can support established PDAC programs [36,37,38,39].	A, M, PF, HO	Opposite stage-linked effects argue against indiscriminate blockade.	Stage-specific hypothesis; interventions remain predominantly preclinical.
TNBC	Sensory nerves interact with PlexinB3; CGRP alters matrix and may contribute to immune exclusion [16,44].	A, M, PF, HO	Neural presence/matrix association does not establish actionable dependency.	Candidate biomarker/mechanism; external validation needed.
Colorectal	Human density/PNI associations and sympathetic degeneration show divergent results; systemic circuits are implicated [20,21,22].	A, M, PF, HO	Studies differ in branch, perturbation breadth, immune context, and endpoint.	Unresolved; no clinical neural intervention established.
Hepatocellular carcinoma	Sympathetic fibers near invasion fronts; catecholamines induce invasive-front programs [23].	A, M, PF, HO	Human association plus cell experiments do not establish clinical causality.	Exploratory; prospective validation absent.
Melanoma	Sympathetic signaling can restrain growth via α2-adrenergic immune effects in mice [24].	M, PF	Preclinical tumor restraint argues against universal neural inhibition.	Preclinical.
Small-cell lung cancer	Peripheral innervation affects primary-tumor development; neuronal activity supports intracranial growth [19].	A, M, PF, HO	Causality is mainly preclinical; peripheral and CNS mechanisms differ.	Emerging preclinical.
Glioma	Neurons provide glutamatergic/cholinergic synaptic input and electrical integration [17,18,26].	A, M, PF, HO	Causal support is stronger in models than for most peripheral tumors.	Advanced model evidence; clinical intervention remains limited.

Note: A, anatomical evidence; M, molecular evidence; PF, preclinical functional evidence; HO, human observational evidence; TNBC, triple-negative breast cancer; PDAC, pancreatic ductal adenocarcinoma. Domains may coexist; the table does not imply prospective biomarker validation or interventional benefit unless explicitly stated.

**Table 5:** Experimental platforms and the strongest defensible claim each platform can support.

Approach	Primary Output	Strongest Defensible Inference	Major Limitation	Recommended Orthogonal Validation
Histology/multiplex IF	Fiber localization and morphology	Neural association and subtype localization [4,34,35]	Limited functional information	Volumetric imaging or functional assay
3D clearing/light-sheet	Branching, volume, trajectory	Network architecture and topology [3,4]	Processing/segmentation artifacts	2D pathology and independent markers
Retrograde tracing/Trace-n-Seq	Projection identity + neuronal state	Identity of tissue-innervating neurons [25,27]	Tracer dependence; mostly preclinical	Functional activity or perturbation
scRNA-seq/snRNA-seq	Cell states and transcriptional programs	Molecular state association [25,28,29,30,31,32,33]	Spatial and axonal context lost	Spatial assay/tracing
Spatial transcriptomics	Molecular geography	Spatial association of states [28,29,30,31,32,33]	Resolution/sensitivity trade-off	High-resolution imaging or in situ validation
MERFISH/in situ imaging	High-resolution target localization	Cellular/subcellular proximity [29]	Targeted panel burden	Orthogonal transcript/protein assays
Organoid/microfluidic co-culture	Dynamic cell–cell interaction	Controlled functional response [26]	Incomplete physiology	In vivo perturbation
Electrophysiology/calcium imaging	Neural and tumor electrical activity	Functional coupling [17,18,26]	Technically demanding	Causal activity manipulation
Optogenetics/chemogenetics	Selective activity manipulation	Causal role of defined neural population [18,19,24,47]	Tool/model specific	Genetic or receptor-level rescue
Denervation	Loss of neural input	Requirement for innervation at the organ level [15,20,21,24]	Injury/systemic confounding	Local-selective perturbation + rescue

**Table 6:** Translational readiness of candidate neural biomarkers and interventions.

Candidate	Current Evidence	Human Validation	Primary Unresolved Issue	Near-Term Development Strategy
Nerve density/subtype	Broad observational + selected preclinical [5,49]	Available in several cancers	Standardization and incremental value	Digital pathology + multicenter validation
Tumor-innervating neuronal signatures	Preclinical projection-defined neuronal signatures; emerging patient relevance [25,46]	Limited	Sampling of distant ganglia	Projection-aware translational profiling
Neural microdomain metrics	Proposed construct; adjacent spatial evidence in neural-invasion profiling [52]	Limited	Assay reproducibility	Standardized spatial pathology pipelines
Adrenergic modulation	Substantial preclinical evidence [1,2,23,24,51,53]	Mixed observational evidence	Confounding and pathway redundancy	Prospective biomarker-selected trials
Muscarinic modulation	Strong gastric/preclinical rationale [15,45]	Limited	Tissue-specific effects	Define receptor-positive populations
Netrin/axon-guidance inhibition	Mechanistic pancreatic evidence [37,39]	Human expression documented	Stage-dependent protective versus tumor-promoting effects	Stage-specific safety and biomarker studies
Sensory-neuropeptide/ECM targeting	Emerging TNBC evidence [44]	Human neural presence documented	Circuit specificity	Spatially selected combination studies
Targeted neuromodulation	Preclinical [49,50]	Very limited oncology evidence	Local control and physiological effects	Focal, reversible, circuit-selective platforms

**Table 7:** Evidence hierarchy for mechanistic and translational claims.

Evidence Level	Question	Appropriate Claim	Overstatement to Avoid
Anatomical	Are neural structures present or enriched?	A neural association is observed	Nerve density proves causality
Molecular	Is a ligand, receptor, or neural state altered?	A candidate pathway or state is implicated	Pathway alteration proves dependence
Spatially resolved	Are specific neural and tumor states co-localized?	A spatial association is supported	Spatial proximity proves signaling
Preclinical functional	Does selective perturbation alter phenotype?	The pathway is functionally relevant in the tested model	The mechanism is clinically validated
Human observational	Does the feature associate with outcome?	The feature may have prognostic relevance	Association demonstrates predictive utility
Prospective biomarker validation	Does the assay improve a defined decision?	Clinical utility is supported	The biomarker is automatically standard of care
Interventional trial	Does targeted neural modulation improve an outcome?	Therapeutic efficacy is supported in the studied population	Preclinical benefit guarantees patient benefit

**Table 8:** Operational distinction between neural abundance, neural state, and neural activity.

Dimension	What Is Measured	Suitable Evidence	What It Cannot Establish by Itself
Neural abundance	Amount and distribution: fiber length/area, density, branches, terminals, subtype density.	Validated histology/IHC or multiplex IF; 2D/3D imaging with stated denominator and sampling.	Does not show neuronal molecular reprogramming, activity, or causal dependence.
Neural state	Identity and molecular program of projection-defined neurons or glia, including RNA/protein state and receptor/neuropeptide repertoire.	Projection-aware tracing with single-cell/single-nucleus profiling; spatial RNA/protein validation.	Does not establish electrical firing, neurotransmitter release, or functional communication.
Neural activity	Time-dependent electrical or chemical output, including spikes, calcium responses, transmitter/neuropeptide release or evoked responses.	Electrophysiology, calcium imaging, direct transmitter assays, time-resolved functional readouts, and controlled perturbation/rescue.	Cannot be inferred from density or transcript abundance alone; activity measurements do not automatically prove tumor dependence.

Neural abundance, neural state, and neural activity are distinct phenotypes and should be reported separately (Table 8).

**Table 9:** Established evidence, emerging findings, and unresolved questions across major cancer–neural contexts.

Cancer/Context	Established Evidence	Emerging Evidence	Unresolved Issue/Decisive Next Step
Pancreatic precursor vs. established PDAC	Netrin-1/DCC-associated sympathetic remodeling in precursor lesions and Netrin-1/NEO1-associated tumor programs in established PDAC have been demonstrated in experimental systems [37,39].	Ganglioside-mediated neural–acinar communication and therapy-associated neural/TLS changes are recent findings [38,40].	When should a pathway be inhibited or preserved? Compare stages, cellular targets and longitudinal immune/tumor phenotypes.
Colorectal cancer	Human nerve-density/PNI associations and experimental effects of neural perturbation are reported [20,21].	Sympathetic degeneration and the preclinical brain–gut circuit broaden the models [21,22].	Use matched immune-competent models, branch-specific perturbation, comparable endpoints and rescue to reconcile divergent directionality.
TNBC/sensory nerves	Sensory-neuron interaction with tumor cells and a CGRP–matrix–immune-exclusion mechanism have experimental support [16,44].	A TLR7-associated innervation signature has been proposed as a patient-stratification candidate [48].	Validate neural signature and immune context prospectively; distinguish prognostic from predictive use.
Melanoma/sympathetic nerves	Experimental evidence supports a tumor-restraining sympathetic/α2-adrenergic immune effect in the tested mouse setting [24].	Translation to human disease and treatment selection remains uncertain.	Determine which patients, disease stages and receptor states preserve or reverse the effect; avoid indiscriminate denervation.
Systemic circuits/metastasis	Local neural effects are demonstrated in selected models, and some tumors integrate with neural circuits.	Brain–gut and tumor–brain–sympathetic circuits are mechanistic preclinical findings [22,47].	Does neural signaling precondition distant sites before arrival, or support cells after colonization? Require longitudinal tracing and perturbation.
Treatment-associated neural remodeling	Surgery and neurotoxic treatments can directly injure nerves, while inflammation, regression and repair can remodel the local tissue.	PDAC nerve-proximal TLS and Schwann-state associations after neoadjuvant treatment are observational [40]; the causal contribution of neural change to treatment response remains unresolved.	Serial paired specimens and function assays must separate direct injury, secondary repair and active tumor–neural adaptation.
Neural biomarkers/intervention	Nerve density and neural signatures may associate with biology or outcomes but are not interchangeable phenotypes.	PDAC TLS and TNBC signatures are candidate biomarkers; perioperative propranolol changed biomarkers but did not establish survival benefit [40,48,51].	Prospective external validation, prespecified intended use, treatment-by-biomarker testing, clinical utility and physiological safety.

## Data Availability

No new data were created or analyzed in this study. Data sharing is not applicable to this article.

## References

[1] Magnon C., Hall S.J., Lin J., Xue X., Gerber L., Freedland S.J., Frenette P.S. (2013). Autonomic nerve development contributes to prostate cancer progression. Science.

[2] Zahalka A.H., Arnal-Estapé A., Maryanovich M., Nakahara F., Cruz C.D., Finley L.W.S., Frenette P.S. (2017). Adrenergic nerves activate an angio-metabolic switch in prostate cancer. Science.

[3] Gillespie S., Monje M. (2020). The neural regulation of cancer. Annu. Rev. Cancer Biol..

[4] Ayala G. (2023). Neuroepithelial interactions in cancer. Annu. Rev. Pathol..

[5] Wang X., Fan Y., Wang Q., Shu X., Lin J., Guo J., Li Z., Xu J. (2025). Tumor-infiltrating nerves: Unraveling the role of cancer neuroscience in tumorigenesis, disease progression, and emerging therapies. Discov. Oncol..

[6] Gu Z., Ma H., Jiang G.Q., Wei J. (2026). Neural regulation of cancer: From microenvironmental hijacking to systemic circuit co-option. Cell. Oncol..

[7] Li X., Li Y., Shi X., Wang Y., Ji S. (2026). Neural regulation of cancer: From synaptic integration to neuro-immune regulation. Cell. Oncol..

[8] Aysit N., Altintas E., Koksalar Alkan F., Ozturk G., Korkaya H. (2026). The neural niche in cancer: Mechanistic insights into tumor-neuron-immune crosstalk and therapeutic opportunities. Front. Cell Dev. Biol..

[9] Magnon C., Hondermarck H. (2023). The neural addiction of cancer. Nat. Rev. Cancer.

[10] Hanahan D., Monje M. (2023). Cancer hallmarks intersect with neuroscience in the tumor microenvironment. Cancer Cell.

[11] Shi D.D., Guo J.A., Hoffman H.I., Su J., Mino-Kenudson M., Barth J.L., Schenkel J.M., Loeffler J.S., Shih H.A., Hong T.S. (2022). Therapeutic avenues for cancer neuroscience: Translational frontiers and clinical opportunities. Lancet Oncol..

[12] Liu Q.Q., Dong Z.K., Wang Y.F., Jin W.L. (2025). Reprogramming neural-tumor crosstalk: Emerging therapeutic dimensions and targeting strategies. Mil. Med. Res..

[13] Wang T., Dong Z., Wang Y., An Z., Wang S., Jin W.L. (2026). Cancer neuroscience: Innovative conception and emerging strategy of therapy. MedComm.

[14] Jin M.Z., Dang H.R., Jin W.L. (2026). Disarming the “neural hijack”: Repurposing neuroactive drugs for cancer therapy. Pharmacol. Ther..

[15] Zhao C.M., Hayakawa Y., Kodama Y., Muthupalani S., Westphalen C.B., Andersen G.T., Flatberg A., Johannessen H., Friedman R.A., Renz B.W. (2014). Denervation suppresses gastric tumorigenesis. Sci. Transl. Med..

[16] Le T.T., Payne S.L., Buckwald M.N., Hayes L.A., Parker S.R., Burge C.B., Oudin M.J. (2022). Sensory nerves enhance triple-negative breast cancer invasion and metastasis via the axon guidance molecule PlexinB3. npj Breast Cancer.

[17] Venkataramani V., Tanev D.I., Strahle C., Studier-Fischer A., Fankhauser L., Kessler T., Körber C., Kardorff M., Ratliff M., Xie R. (2019). Glutamatergic synaptic input to glioma cells drives brain tumour progression. Nature.

[18] Venkatesh H.S., Morishita W., Geraghty A.C., Silverbush D., Gillespie S.M., Arzt M., Tam L.T., Espenel C., Ponnuswami A., Ni L. (2019). Electrical and synaptic integration of glioma into neural circuits. Nature.

[19] Savchuk S., Gentry K.M., Wang W., Carleton E., Biagi-Junior C.A.O., Luthria K., Yalçın B., Ni L., Farnsworth H.C., Davis R.A. (2025). Neuronal activity-dependent mechanisms of small cell lung cancer pathogenesis. Nature.

[20] Wang H., Huo R., He K., Li W., Gao Y., He W., Yu M., Jiang S.-H., Xue J. (2025). Increased nerve density adversely affects outcome in colorectal cancer and denervation suppresses tumor growth. J. Transl. Med..

[21] Chen X., Cao Y., Zhao Y., Ma Y., Shi X., Wang J., Jiang Z., Luo R., Deng Z., Zhou X. (2025). Neurodegeneration of local sympathetic inputs promotes colorectal cancer progression. Cancer Lett..

[22] Li Y., Yu H., Li Z.M., Yin K.-W., Jin S.-Y., Chen C.-C., Tan M.-S., Zhang C.-J., Liu X.-H., Li W.-P. (2025). Colorectal cancer cells hijack a brain-gut polysynaptic circuit from the lateral septum to enteric neurons to sustain tumor growth. Nat. Cancer.

[23] Lelou E., Rauch C., Dubois-Pot-Schneider H., Dufresne M., Yazdi A.Z., Guillout M., Cano L., Desquilles L., Nesseler N., Bourgeois M. (2026). Catecholamines activate a tumour progression program in differentiated tumour hepatocytes. Liver Int..

[24] Liu T., Kutsovsky D.Y., Earlie E.M., Ji L., Iskols M., Ramsamooj S., Dawkins X.I., Zerhouni M., Birbrair A., Piskounova E. (2026). A local sympathetic-immune axis inhibits melanoma growth in mice by dictating adrenergic control. Neuron.

[25] Thiel V., Renders S., Panten J., Dross N., Bauer K., Azorin D., Henriques V., Vogel V., Klein C., Leppä A.-M. (2025). Characterization of single neurons reprogrammed by pancreatic cancer. Nature.

[26] Sun Y., Wang X., Zhang Z., Park K.H., Wu Y., Dong W., Zhang D.Y., Fu Y., Zhang F., Binder Z.A. (2025). Cholinergic neuron-to-glioblastoma synapses in a human iPSC-derived co-culture model. Stem Cell Rep..

[27] Sun Y., Wang X., Zhang Z., Ming G.L., Song H. (2026). Transsynaptic tracing techniques to interrogate neuronal connectivity of glioblastomas. Nat. Protoc..

[28] Stahl P.L., Salmen F., Vickovic S., Lundmark A., Navarro J.F., Magnusson J., Giacomello S., Asp M., Westholm J.O., Huss M. (2016). Visualization and analysis of gene expression in tissue sections by spatial transcriptomics. Science.

[29] Chen K.H., Boettiger A.N., Moffitt J.R., Wang S., Zhuang X. (2015). RNA imaging. Spatially resolved, highly multiplexed RNA profiling in single cells. Science.

[30] Rodriques S.G., Stickels R.R., Goeva A., Martin C.A., Murray E., Vanderburg C.R., Welch J., Chen L.M., Chen F., Macosko E.Z. (2019). Slide-seq: A scalable technology for measuring genome-wide expression at high spatial resolution. Science.

[31] Stickels R.R., Murray E., Kumar P., Li J., Marshall J.L., Di Bella D.J., Arlotta P., Macosko E.Z., Chen F. (2021). Highly sensitive spatial transcriptomics at near-cellular resolution with Slide-seqV2. Nat. Biotechnol..

[32] Cilento M.A., Sweeney C.J., Butler L.M. (2024). Spatial transcriptomics in cancer research and potential clinical impact: A narrative review. J. Cancer Res. Clin. Oncol..

[33] Mo J., Bae J., Saqib J., Hwang D., Jin Y., Park B., Park J., Kim J. (2024). Current computational methods for spatial transcriptomics in cancer biology. Adv. Cancer Res..

[34] Demir I.E., Boldis A., Pfitzinger P.L., Teller S., Brunner E., Klose N., Kehl T., Maak M., Lesina M., Laschinger M. (2014). Investigation of Schwann cells at neoplastic cell sites before the onset of cancer invasion. J. Natl. Cancer Inst..

[35] Deborde S., Omelchenko T., Lyubchik A., Zhou Y., He S., McNamara W.F., Chernichenko N., Lee S.-Y., Barajas F., Chen C.-H. (2016). Schwann cells induce cancer cell dispersion and invasion. J. Clin. Investig..

[36] Stopczynski R.E., Normolle D.P., Hartman D.J., Ying H., DeBerry J.J., Bielefeldt K., Rhim A.D., DePinho R.A., Albers K.M., Davis B.M. (2014). Neuroplastic changes occur early in the development of pancreatic ductal adenocarcinoma. Cancer Res..

[37] Haidar H., Bellon A., Sleiman K., Hocine M., Rama N., Gadot N., Carpizo D.R., Mehlen P., Mann F. (2025). Neural function of Netrin-1 in precancerous lesions of the pancreas. Nat. Commun..

[38] Wei F., Zou Q., An J., Zhang T., Sun Q., Li X., Jiang T., Zhu X., Wen T., He J. (2026). Ganglioside-mediated neural-acinar crosstalk facilitates pancreatic carcinogenesis via metaplastic niche remodeling. Gastroenterology.

[39] Kobayashi H., Ochiai Y., Arai J., Hata M., Wu F., Sunagawa M., Iida T., Baba T., Malagola E., Tanaka T. (2026). Netrin-1 promotes pancreatic tumorigenesis and innervation through NEO1. Cancer Res..

[40] Cai S., Yang M.W., Jiang L., Weng Z., Wang X., Ma X., Huo Y.-M., Liu W., Zhang S., Jiang S.-H. (2026). Nerve-proximal tertiary lymphoid structures predict chemotherapy sensitivity in pancreatic cancer. Cell Rep..

[41] Thielman N.R.J., Funes V., Davuluri S., Ibanez H.E., Sun W.C., Fu J., Li K., Muth S., Pan X., Fujiwara K. (2024). Semaphorin 3D promotes pancreatic ductal adenocarcinoma progression and metastasis through macrophage reprogramming. Sci. Adv..

[42] Banh R.S., Biancur D.E., Yamamoto K., Sohn A.W.S., Walters B., Kuljanin M., Gikandi A., Wang H., Mancias J.D., Schneider R.J. (2020). Neurons release serine to support mRNA translation in pancreatic cancer. Cell.

[43] Madeo M., Colbert P.L., Vermeer D.W., Lucido C.T., Cain J.T., Vichaya E.G., Grossberg A.J., Muirhead D., Rickel A.P., Hong Z. (2018). Cancer exosomes induce tumor innervation. Nat. Commun..

[44] Zhang S.W., Wang H., Xiao Y., Liu L.T., Shen M., Wang Z., Zhao S., Ding X.-H., Wang Y., Zhuang Q.-Y. (2026). Sensory neurons drive immune exclusion by stimulating a dense extracellular matrix in the breast cancer tumor microenvironment. Cell.

[45] Hayakawa Y., Sakitani K., Konishi M., Asfaha S., Niikura R., Tomita H., Renz B.W., Tailor Y., Macchini M., Middelhoff M. (2017). Nerve growth factor promotes gastric tumorigenesis through aberrant cholinergic signaling. Cancer Cell.

[46] Amit M., Takahashi H., Dragomir M.P., Lindemann A., Gleber-Netto F.O., Pickering C.R., Anfossi S., Osman A.A., Cai Y., Wang R. (2020). Loss of p53 drives neuron reprogramming in head and neck cancer. Nature.

[47] Wei H.K., Yu C.D., Hu B., Zeng X., Ichise H., Li L., Wang Y., Wang R.L., Germain R.N., Chang R.B. (2026). Tumour-brain crosstalk restrains cancer immunity via a sensory-sympathetic axis. Nature.

[48] Wang D.Y., Jiang Z., Ben-David Y., Done S.J., Zacksenhaus E. (2026). TLR7 signature of tumour innervation reveals two distinct pathways of triple-negative breast cancer progression. Br. J. Cancer.

[49] Yaniv D., Mattson B., Talbot S., Gleber-Netto F.O., Amit M. (2024). Targeting the peripheral neural-tumour microenvironment for cancer therapy. Nat. Rev. Drug Discov..

[50] Yuan M., Xi R., Kang Y., Kuang M.J., Ji X. (2025). Cancer neuroscience: Decoding neural circuitry in tumor evolution for targeted therapy. Adv. Sci..

[51] Hiller J.G., Cole S.W., Crone E.M., Byrne D.J., Shackleford D.M., Pang J.M., Henderson M.A., Nightingale S.S., Ho K.M., Myles P.S. (2020). Preoperative beta-blockade with propranolol reduces biomarkers of metastasis in breast cancer: A phase II randomized trial. Clin. Cancer Res..

[52] Chen M.M., Gao Q., Ning H., Chen K., Gao Y., Yu M., Liu C.-Q., Zhou W., Pan J., Wei L. (2025). Integrated single-cell and spatial transcriptomics uncover distinct cellular subtypes involved in neural invasion in pancreatic cancer. Cancer Cell.

[53] Partecke L.I., Speerforck S., Kading A., Seubert F., Kuhn S., Lorenz E., Schwandke S., Sendler M., Keßler W., Trung D.N. (2016). Chronic stress increases experimental pancreatic cancer growth, reduces survival and can be antagonised by beta-adrenergic receptor blockade. Pancreatology.

[54] Chen G., Lu W., Liu M., Ye Q., Xu Y., Zhang Y., Guo X., Ye Y., Yang X., Luo X. (2026). Tumor-derived GDF15 induces CCN3+ Schwann cells to promote cancer pain in pancreatic cancer. Nat. Commun..

[55] O’Logbon J., Tarantola L., Williams N.R., Mehta S., Ahmed A., Davies E.A. (2025). Does propranolol have a role in cancer treatment? A systematic review of the epidemiological and clinical trial literature on beta-blockers. J. Cancer Res. Clin. Oncol..

[56] Jones G., Anderson J.L., Nguyen P.T.T., Gleber-Netto F.O., Hwang W.L., Puduvalli V.K., Monje M., Amit M. (2025). Novel approaches to clinical trial design in cancer neuroscience. Neuron.

[57] Zhao J., Cheng L., Yang J., Xu F., Qi W., Liao K., Zhou L., Cao L., Chen J., Lin Y. (2025). Tumor-nerve interactions in cancer regulation and progression. Cancer Lett..

[58] Huang Q., Hu B., Zhang P., Yuan Y., Yue S., Chen X., Liang J., Tang Z., Zhang B. (2025). Neuroscience of cancer: Unraveling the complex interplay between the nervous system, the tumor and the tumor immune microenvironment. Mol. Cancer.

